# Scaphotrapeziotrapezoid Arthrodesis Using Limited Wrist Fusion Plates in Kienböck’s Disease

**DOI:** 10.7759/cureus.4025

**Published:** 2019-02-07

**Authors:** Baver Acar, Adil Turan, Ozkan Kose, Serra Ozturk, Muzaffer Sindel

**Affiliations:** 1 Orthopaedics, University of Health Sciences, Antalya Education and Research Hospital, Antalya, TUR; 2 Anatomy, Akdeniz University, Antalya, TUR

**Keywords:** kienböck’s disease, scaphotrapeziotrapezoid arthrodesis, limited wrist fusion, plate fixation

## Abstract

Purpose: The purpose of this study was to evaluate the clinical and radiological results of scaphotrapeziotrapezoid (STT) arthrodesis with a limited wrist fusion plate in patients with Stage IIIB Kienböck’s disease (KD).

Materials and methods: A retrospective review was performed on nine patients with Stage IIIB KD who underwent STT arthrodesis between 2014 and 2017 at our institution. Clinical evaluations of the patients were made using the shortened quick version of the Disabilities of the Arm, Shoulder, and Hand (Q-DASH) Outcome Measure score (Institute for Work and Health, Toronto, ON, Canada) and grip strength measurements before surgery and at the final follow-up examination. All patients underwent computed tomography (CT) scan to confirm the union of the arthrodesis.

Results: A complete union was obtained in all patients. The Q-DASH score was changed from 57.8 ± 8.2 points (range: 47.7 - 70.5) to 32.3 ± 17.3 points (range: 13.6 - 54.5) (p = 0.008). Similarly, the grip strength was improved significantly (p = 0.007).

Conclusions: The use of limited wrist fusion plates for STT arthrodesis in KD is a safe and effective treatment method that provides a high rate union and acceptable functional results.

## Introduction

Kienböck’s disease (KD), also called as avascular necrosis of the lunate, is a rare wrist disorder characterized by progressive osteonecrosis of the lunate. Robert Kienböck first defined the radiological characteristics in 1910 [1]. The incidence in the general population has been reported between 0.0066% and 0.27% in radiographic survey studies [2-3]. It is commonly seen in males aged 20 to 40 years. Occasionally, it is bilateral, and there is often a history of previous trauma. Although there are radiological findings of destruction in the lunate bone in all patients, some cases may also be asymptomatic, despite positive radiographic findings [2, 4].

The exact etiology of the disease is uncertain. However, various anatomic and biomechanical risk factors are thought to play a role in the pathophysiology. Ulnar negative variance (causing an increase in radio-lunate contact stress), reduced radial inclination, repetitive wrist trauma, the geometry of the lunate bone, and the interruption of the vascular supply of the lunate bone are among the most commonly blamed risk factors cited in the current literature [5-6].

The Lichtman classification is the most commonly used staging system that separates KD into four stages based on radiographic findings [7]. According to the Lichtman classification, Stage IIIB is an advanced stage that is defined as a lunate collapse with fixed scaphoid rotation (ring sign), decreased carpal height, and proximal migration of the capitate. Various surgical methods have been recommended at this stage, including limited carpal fusion, vascularized pedicled bone grafts, proximal row carpectomy (PRC), and the use of a bone morphogenic protein with an arthroscopic technique. However, there is no reliable evidence supporting the superiority of any of these surgical techniques over the others [8-9]. Scaphotrapeziotrapezoid (STT) fusion has been shown to provide good results in KD. It is thought that arthrodesis could reduce the progression of necrosis by decreasing the load on the lunate bone and preventing movement between the lunate bone and the distal row of the carpal bones [10].

STT arthrodesis can be performed using various fixation implants or techniques. To the best of our knowledge, there is no study related to the use of a limited wrist fusion plate in KD in the current literature. This study aimed to evaluate the clinical and radiological results of STT arthrodesis with a limited wrist fusion plate in patients with Stage IIIB KD.

## Materials and methods

A retrospective review was performed on 11 patients with KD who underwent STT arthrodesis between 2014 and 2017 in our institution. The radiological images were retrieved from the picture archiving and communication system (PACS), and the demographic data and clinical findings were obtained from the patient charts, medical records, operation notes, and follow-up notes in the institutional patient database. Of the original 11 patients, two were lost during follow-up. Thus, the study was completed with the remaining nine patients who completed the follow-up examinations.

This study was conducted in accordance with the ethical standards laid down in the 1964 Declaration of Helsinki and its later amendments. The Institutional Review Board (IRB) of Akdeniz University approved the study protocol, and all participants provided informed consent for inclusion in the study.

**Figure 1:**
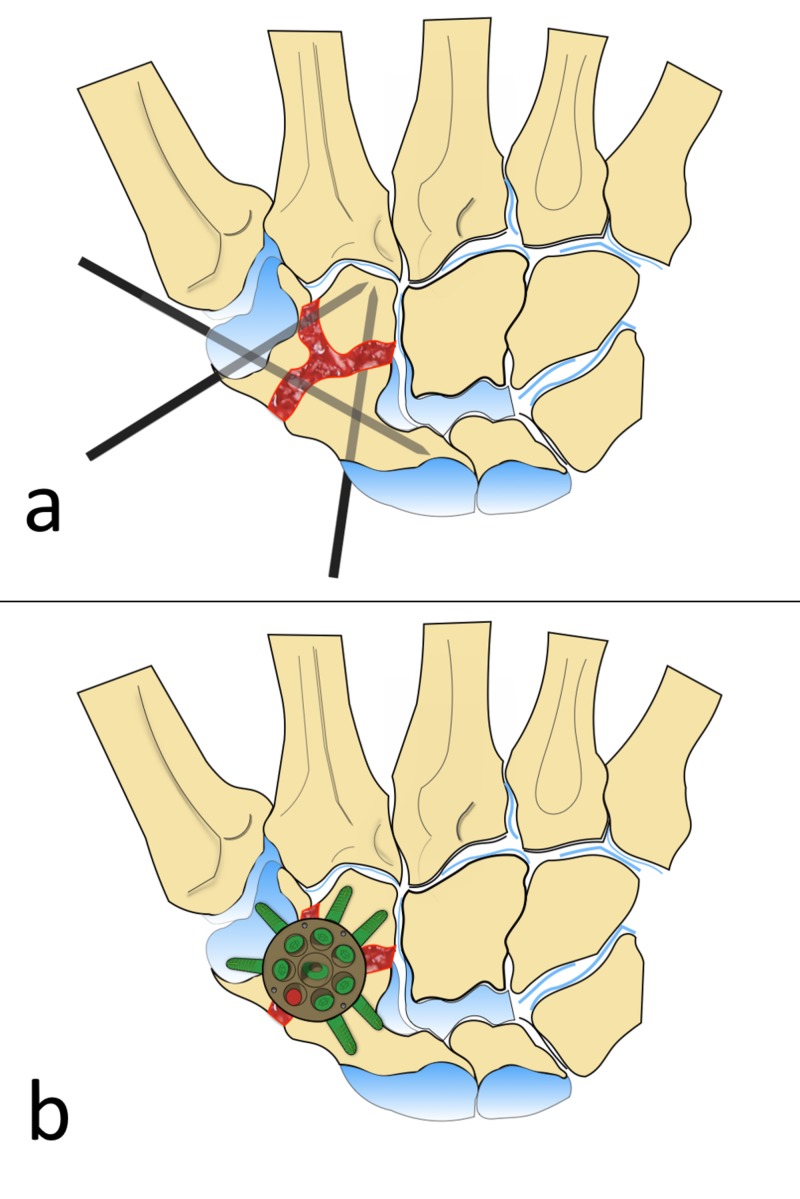
Illustration Showing the Surgical Technique A) Cartilage at the articular surfaces of STT joints was carefully removed, autologous cancellous bone grafting was placed at the arthrodesis site, and three provisional K-wires were used to fix the STT joint in the desired position; B) A special reamer was used to prepare the bed for the limited wrist fusion plate, which was then placed at the center of the STT joint, and each carpal bone was fixed with two screws whenever possible. STT: scaphotrapeziotrapezoid The illustrations were drawn by Adil Turan, M.D.

All the patients were operated by the senior author (BA). Under general anesthesia and with the use of a tourniquet, a dorsal longitudinal incision was made over the wrist in line with the STT joint. The extensor tendons were retracted, and the wrist joint capsule was reached. Following capsulotomy, all cartilage in the arthrodesis area (STT joints) was carefully removed using a high-speed burr and curettes until optimal cancellous bone contact for fusion was obtained. Autologous cancellous bone graft was harvested from the distal radius and placed in the arthrodesis site. Two or three temporary K-wires were used to fix the STT joint in the desired position (Figure 1A). A special reamer was used to prepare the bed for the limited wrist fusion plate (Mini Hub Cap®, Acumed, LLC, Hillsboro, OR, USA), which was then placed at the center of the STT joint; each carpal bone was fixed with two screws whenever possible (Figure 1B). The plate and the screws were checked under fluoroscopy. The capsule and retinaculum were sutured and then the skin was closed. A short-arm spica cast was applied to the patient.

The wrist was immobilized for four weeks, after which a three-week physiotherapy program was started, including passive and active finger and wrist range of motion exercises. All patients were given a home-based physical therapy program.

Anteroposterior and lateral wrist radiographs were taken, and magnetic resonance imaging (MRI) was performed on the wrist of all patients. The Litchman classification was used to evaluate the KD stage [7-8]. All patients attended regular follow-up examinations with evaluation of the union from anteroposterior and lateral wrist radiographs. In the sixth month, all patients underwent computed tomography (CT) scanning to confirm the complete union and consolidation of the arthrodesis. 

Clinical evaluations of the patients were made using the shortened quick version of the Disabilities of the Arm, Shoulder, and Hand (Q-DASH) Outcome Measure score (Institute for Work and Health, Toronto, ON, Canada), as well as grip strength measurements before surgery and at the final follow-up examination. Grip strength measurements were taken using a calibrated hydraulic hand dynamometer (Base­line®, Fabrication Enterprises, Inc., White Plains, NY, USA). All measurements were made in accordance with the standardized instructions of the American Society for Surgery of the Hand and the American Society of Hand Therapists [11]. Wrist range of motion was measured using a goniometer. To assess overall subjective patient satisfaction, patients were asked if they would have the same treatment again at the final follow-up examination.

Continuous variables were stated as mean ± standard deviation values and categorical variables as number (n) and percentage (%). The comparison of serial measurements within the same group was performed using the Wilcoxon signed-rank test. A value of p < 0.05 was considered statistically significant.

## Results

The patients in this study comprised six males and three females with a mean age of 32.8 ± 7 years (range: 22 - 44). All patients were right-handed, and the operation was on the dominant side in six cases. According to the Lichtman classification, all patients were at Stage III B. The mean follow-up duration was 18.4 ± 10.8 months (range: 8 - 36). The demographic and clinical characteristics are presented in Table 1.

**Table 1:** Demographic and Clinical Characteristics of Patients F: female; L: left; M: male; R: right

Case #	Age	Sex	Side	Dominancy	Stage	Occupation
1	38	F	L	R	III B	Housewife
2	23	M	R	R	III B	Teacher
3	44	F	R	R	III B	Farmer
4	35	M	R	R	III B	Officer
5	37	F	L	R	III B	Housewife
6	32	M	R	R	III B	Officer
7	35	M	L	R	III B	Heavy worker
8	30	M	R	R	III B	Officer
9	22	M	R	R	III B	Farmer

The Q-DASH score was changed from 57.8 ± 8.2 points (range: 47.7 - 70.5) to 32.3 ± 17.3 points (range: 13.6 - 54.5) (p = 0.008). Similarly, the grip strength was improved significantly (p = 0.007). However, compared to the contralateral side, the grip strength was still weak (p = 0.008). Clinical results are summarized in Table 2.

**Table 2:** Summary of Clinical Outcomes Q-DASH: shortened quick version of the Disabilities of the Arm, Shoulder, and Hand

Case #	Follow-up (months)	Q-DASH (points)		Grip Strength (kg)		
		Preoperative	Postoperative	Preoperative	Postoperative	Contralateral side
1	34	61.4	45.45	22	26	33
2	26	50	25	15	20	37.2
3	11	59.1	13.63	16	19.3	23.3
4	18	47.7	18.18	24	31.3	38.3
5	36	52.3	13.63	19	20.6	26
6	13	68.2	54.54	12	16	30
7	10	61.4	47.72	20	24	34
8	10	70.5	52.27	16	21	38.3
9	8	50	20.45	17	20.6	36

**Figure 2:**
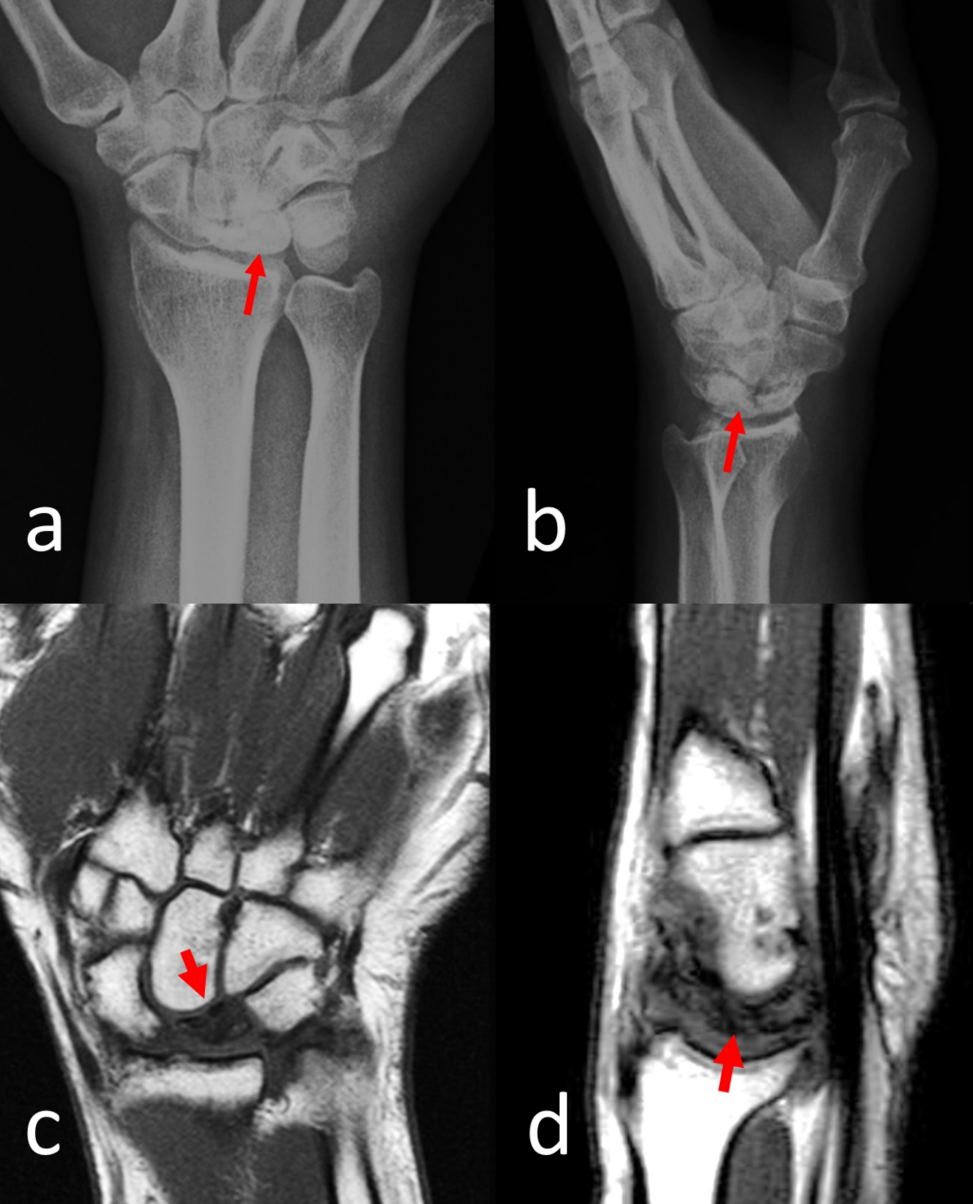
Preoperative Imaging of Case 4 A) anteroposterior and B) lateral wrist radiographs showing Stage IIIB Kienböck’s disease. The red arrows show the dense sclerotic lunate in direct radiographs. C) coronal and D) sagittal T1-weighted magnetic resonance imaging of the patient showed avascular necrosis of the lunate bone. Note the low signal intensity of the lunate demonstrating avascular necrosis (red arrows).

**Figure 3:**
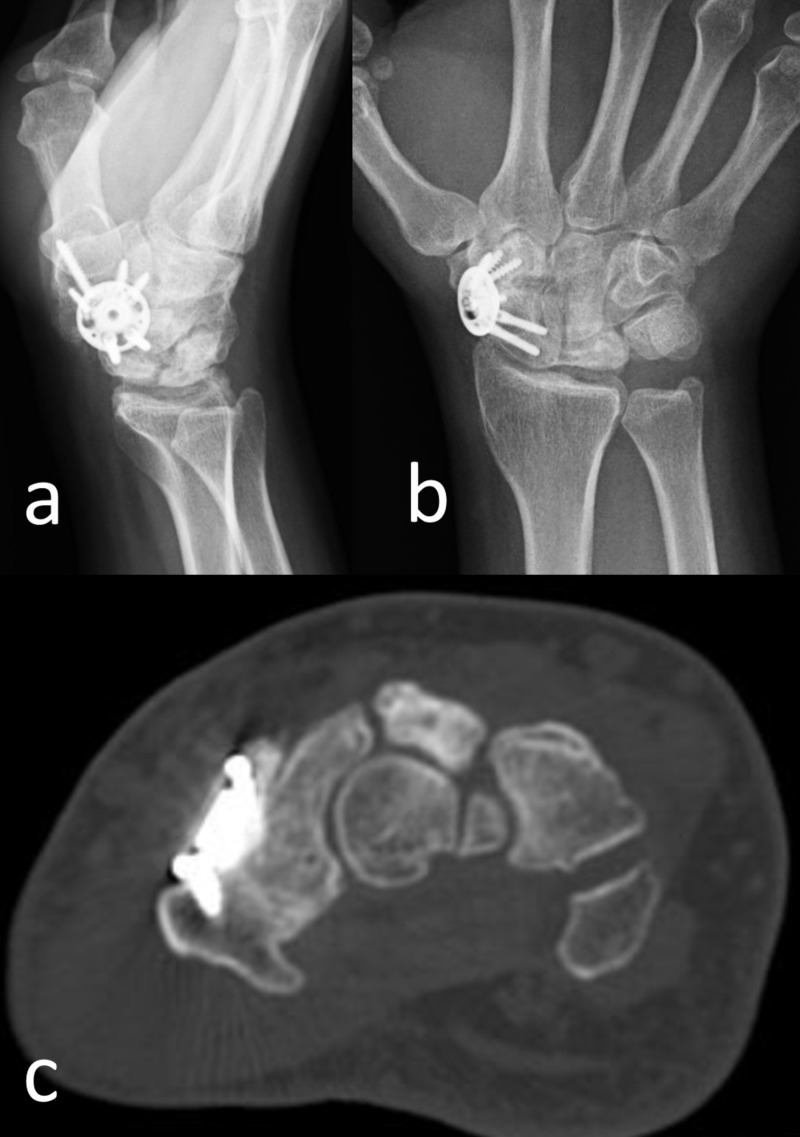
Postoperative Imaging of Case 4 A) Lateral and B) anteroposterior wrist radiographs at the final follow-up at the 18th postoperative month. C) computed tomography examination demonstrated complete union and consolidation of the arthrodesis.

At the final follow-up evaluation, the wrist motion was restricted compared to the contralateral side in all directions (flexion, extension, ulnar, and radial deviation). All patients were free of night pain and were satisfied with the overall treatment. CT examination showed the complete union of the arthrodesis in all patients (Figures 2-3). The average duration of time to union was 4.6 months. No complication was seen in any patient.

## Discussion

In this study, STT arthrodesis using limited wrist fusion plate was evaluated for the treatment of KD. Postoperatively, night pain and pain at rest was seen to have recovered in all the patients. No complications, such as non-union or infection, developed in any patient. All the patients reported that they had no pain that would prevent work. Union was observed in all patients within an accepted time interval. Functional scores increased significantly in all patients, and all patients were satisfied with the treatment. Overall, we think that the limited wrist fusion plate is an appropriate implant selection in patients with KD considered to have STT arthrodesis.

There is no gold standard in treating Stage IIIB KD to date. PRC, STT arthrodesis, and scaphocapitate (SC) arthrodesis remain the most commonly used surgical techniques in Stage IIIB KD. In PRC, the scaphoid, lunate, and triquetrum are excised, and the load on the wrist is carried over the capitate and lunate fossa. After performing PRC, Richou et al. reported 83% satisfaction of the patients in their series of 24 patients of which 17 were KD. They reported that the pain was reduced in all patients, the grip strength reached 76% of the contralateral side, the wrist flexion-extension arc was 76°, and the mean DASH score was 31 points [12]. Similarly, Croog and Stern reported a low complication rate, painless movement, and adequate grip strength after PRC in 18 patients who were followed with a mean of 10 years [13].

STT arthrodesis stabilizes the scaphoid in the radial fossa, absorbs the load, and prevents advanced scapholunate collapse. Watson et al. reported the results of 28 patients treated with STT arthrodesis in KD after a mean follow-up of 51 months. The functional outcomes were excellent in 12 patients, good in nine, fair in four, and poor in two patients in their series [14]. SC arthrodesis is another option for Stage IIIB KD disease. The SC joint has a larger bone contact surface compared to the STT joint. Furthermore, only two carpal bones are involved in the arthrodesis. Although reliable pain relief usually occurs with SC fusion, patients display less midcarpal mobility (e.g., dart-throwing motion) than STT arthrodesis [15-16].

K-wires, U-nails, or headless compression screws can be used in wrist-limited fusion surgery. Although K-wires are inexpensive, they do not provide compression at the arthrodesis interface. Similarly, U-nails cannot exert the desired compression, and after the application of K-wires or U-nails, the wrist must be immobilized with a plaster cast until sufficient fusion is achieved to allow wrist motion. Because these implants lack compression properties, nonunion is a common complication after K-wire and U-nail fixation. Fortin et al. reported a 21% nonunion rate in STT arthrodesis (three of 14 patients) performed with K-wires [17]. Kleinman retrospectively evaluated 46 patients with wrist instability who underwent STT arthrodesis using K wires and reported 8% (four patients) with pin tract infection, 4% (two patients) with infection, and 15% (eight patients) with nonunion [18]. Similarly, Frykman et al. reported a 29% nonunion rate in five out of 17 patients who underwent STT arthrodesis due to scaphoid instability and KD using K wires and U staples [19]. All these previous findings confirm that implants that do not provide compression and stable fixation are associated with a high rate of nonunion and complications. On the other hand, compression can be implemented with variable-pitched cannulated screws, such as Herbert screws. However, proper placement and positioning of these implants (perpendicular to the arthrodesis interface) are difficult due to the surrounding carpal bones, and the learning curve is long [20]. The high prevalence of nonunion seen in carpal arthrodesis necessitated a more stable fixation, and subsequently, limited wrist fusion plates were introduced in 1999 [21].

Limited wrist fusion plates are mainly used in the four-corner carpal fusion in scapholunate advanced collapse (SLAC) and scaphoid nonunion advanced collapse (SNAC) wrist diseases. Successful results have been reported after the use of these implants [22]. Bedford reported 100% union in a series of 15 patients with these plates in four-corner fusion [21]. Similarly, Leugmair performed four-corner fusion to a series of 24 patients and reported union in 22 (91%) [23]. In the current study, the 100% union was achieved with limited wrist fusion plates. Although these plates do not provide compression, each carpal bone is fixed with two screws which provide strong stability. We believe that stability is another crucial requirement for gaining union. This may be an explanation for the high rate of union reported in the literature and our study.

It is noteworthy to mention that there are some limitations to this study. The inclusion of a limited number of patients and short follow-up duration are primary limitations. KD is a rare disease, and only the Stage IIIB KD patients were included in the study. Since a subgroup of a rare disease is studied, the number of patients was low. However, this is the first study that reported the use of limited wrist fusion plate in KD in current literature. Moreover, the bone union was assessed with CT in all the patients.

## Conclusions

This study investigated the use of limited wrist fusion plates for STT arthrodesis in KD. Intercarpal fusion was achieved in all the patients with improvement in functional outcomes. We believe that biomechanical stability provided by the plate and the locking screws is the main reason behind this high rate of union. Use of limited wrist fusion plates is a safe and effective treatment method for STT arthrodesis. Future studies which compare different fixation techniques in STT arthrodesis will elucidate the optional fixation choice.

## References

[1] Peltier LF (1980). The classic. Concerning traumatic malacia of the lunate and its consequences: degeneration and compression fractures. Privatdozent Dr Robert Kienböck. Clin Orthop Relat Res.

[2] Golay SK, Rust P, Ring D (2016). The radiological prevalence of incidental Kienböck disease. Arch Bone Jt Surg.

[3] van Leeuwen WF, Janssen SJ, ter Meulen DP, Ring D (2016). What is the radiographic prevalence of incidental Kienböck disease?. Clin Orthop Relat Res.

[4] Lichtman DM, Mack GR, MacDonald RI, Gunther SF, Wilson JN (1977). Kienböck's disease the role of silicone replacement arthroplasty. J Bone Joint Surg Am.

[5] Iwasaki N, Genda E, Minami A, Kaneda K, Chao EY (1998). Force transmission through the wrist joint in Kienböck's disease: a two-dimensional theoretical study. J Hand Surg Am.

[6] Iwasaki N, Genda E, Barrance PJ, Minami A, Kaneda K, Chao EY (1998). Biomechanical analysis of limited intercarpal fusion for the treatment of Kienböck's disease: a three-dimensional theoretical study. J Orthop Res.

[7] Allan CH, Joshi A, Lichtman DM (2001). Kienböck’s disease: diagnosis and treatment. J Am Acad Orthop Surg.

[8] Rhee SK, Kim HM, Bahk WJ, Kim YW (1996). A comparative study of the surgical procedures to treat advanced Kienbock's disease. J Korean Med Sci.

[9] Nakamura R, Horii E, Watanabe K, Nakao E, Kato H, Tsunoda K (1998). Proximal row carpectomy versus limited wrist arthrodesis for advanced Kienböck's disease. J Hand Surg Br.

[10] Meier R, van Griensven M, Krimmer H (2004). Scaphotrapeziotrapezoid (STT)-arthrodesis in Kienböck’s disease. J Hand Surg Br.

[11] Unal M, Kose O, Arik HO, Guler F, Acar B, Yuksel HY (2018). Hand grip strength: age and gender stratified normative data in Anatolian population. Hand Microsurg.

[12] Richou J, Chuinard C, Moineau G, Hanouz N, Hu W, Le Nen D (2010). Proximal row carpectomy: long-term results. Chir Main.

[13] Croog AS, Stern PJ (2008). Proximal row carpectomy for advanced Kienböck's disease: average 10-year follow-up. J Hand Surg Am.

[14] Oishi SN, Muzaffar AR, Carter PR (2002). Treatment of Kienbock's disease with capitohamate arthrodesis: pain relief with minimal morbidity. Plast Reconstr Surg.

[15] Pisano SM, Peimer CA, Wheeler DR, Sherwin F (1991). Scaphocapitate intercarpal arthrodesis. J Hand Surg Am.

[16] Sennwald GR, Ufenast HJ (1995). Scaphocapitate arthrodesis for the treatment of Kienböck's disease. J Hand Surg Am.

[17] Fortin PT, Louis DS (1993). Long-term follow-up of scaphoid-trapezium-trapezoid arthrodesis. J Hand Surg Am.

[18] Kleinman WB, Carroll C 4th (1990). Scapho-trapezio-trapezoid arthrodesis for treatment of chronic static and dynamic scapho-lunate instability: A 10-year perspective on pitfalls and complications. J Hand Surg Am.

[19] Frykman EB, Af Ekenstam F, Wadin K (1988). Triscaphoid arthrodesis and its complications. J Hand Surg Am.

[20] Ledgard JP, Siddiqui J, Pelletier MH, Walsh WR, Scougall PJ (2018). Midcarpal arthrodesis biomechanics memory staples versus cannulated screws. J Hand Surg Asian Pac.

[21] Bedford B, Yang SS (2010). High fusion rates with circular plate fixation for four-corner arthrodesis of the wrist. Clin Orthop Relat Res.

[22] Woehl R, Maier J, Gehmert S, Palm C, Riebschlaeger B, Nerlich M, Huber M (2018). 3D analysis of Osteosyntheses material using semi-automated CT segmentation: a case series of a 4 corner fusion plate. BMC Musculoskelet Disord.

[23] Luegmair M, Houvet P (2012). Effectiveness of four-corner arthrodesis with use of a locked dorsal circular plate. Clin Orthop Relat Res.

